# Research on the Mechanism of HRP Relieving IPEC-J2 Cells Immunological Stress Based on Transcriptome Sequencing Analysis

**DOI:** 10.3389/fnut.2022.944390

**Published:** 2022-07-15

**Authors:** Muyang Li, Lu Chen, Yiran Zhao, Hui Sun, Lei Zhao

**Affiliations:** ^1^College of Animal Science and Veterinary Medicine, Heilongjiang Bayi Agricultural University, Daqing, China; ^2^Shanxi Animal Husbandry and Veterinary School, Taiyuan, China; ^3^College of Food Science, Heilongjiang Bayi Agricultural University, Daqing, China; ^4^College of Animal Science and Technology, Jilin Agricultural University, Changchun, China

**Keywords:** IPEC-J2 cells, HRP, anti-inflammatory, MAPK/NF-κB signaling pathway, transcriptome

## Abstract

Early weaning increased the economic benefits of piglets. However, early weaning damages the intestinal barrier of piglets and causes immunological stress. The mechanism by which *Hippophae rhamnoides* polysaccharide (HRP) alleviates lipopolysaccharide (LPS)-induced intestinal porcine epithelial cells (IPEC-J2) inflammatory damage was investigated using proteomics in our previous studies. In this study we employed RNA-sequencing (RNA-seq) to determine the level and function of differentially expressed genes (DEGs) and further explore the mechanism of the HRP anti-inflammatory and immune process. The differential expression analysis indicated that 3622, 1216, and 2100 DEGs in the IPEC-J2 cells were identified in C vs. L, L vs. H6-L, and C vs. H6-L, respectively. The Kyoto Encyclopedia of Genes and Genomes (KEGG) enrichment analysis foundsix identified pathways related to the immune system. Additionally, we used the Science, Technology, Engineering, and Math (STEM) program to categorize the 3,134 DEGs that were differentially expressed in H2-L, H4-L and H6-L into eight possible expression profiles, in which 612 were clustered into two profiles. The accuracy and consistency of RNA-seq data were validated by the results of qRT-PCR of the nuclear factor of kappa light polypeptide gene enhancer in B-cells 2 (NFKB2), MAP kinase interacting serine/threonine kinase 2 (MKNK2), mitogen-activated protein kinase kinase 1 (MAP2K1), mitogen-activated protein kinase kinase kinase 8 (MAP3K8), Ras-related protein R-Ras (RRAS), TNF receptor-associated factor 1 (TRAF1), NF-kappa-B inhibitor alpha (NFKBIA), interleukin 8 (IL8), tumor necrosis factor, alpha-induced protein 3 (TNFAIP3), and transforming growth factor beta-1 (TGFB1). Transcriptome sequencing also indicated that HRP reduced the expression levels of related DEGs and inhibited the activation of the mitogen-activated protein kinase (MAPK)/nuclear factor kappa-B (NF-κB) signaling pathway. Our findings indicate that the application of HRP in piglet diets during the early weaning period can improve intestinal epithelial function and integrity, and relieve intestinal damage, and improve piglet health.

## Introdution

With the acceleration of the large-scale and intensified process of pig production, early weaning techniques for piglets have been gradually implemented (1). Weaned piglets are affected by stresses such as nutrition, immunity, and environment, and piglets have early weaning syndromes such as reduced feed utilization, poor growth, and diarrhea (2, 3). Piglet weaning is accompanied by the occurrence of intestinal inflammation, which causes a series of negative reactions in the incompletely developed intestinal tracts of piglets, such as intestinal mucosal injury, intestinal villi damage, and intestinal wall injury, Intestinal inflammation also affects the digestive and absorptive function of the intestinal tract in piglets and leads to sluggish growth, diarrhea, and even death, causing great economic losses to the swine industry (4). The intestinal mucosa is the host's first line of defense against pathogenic microorganisms. Intestinal epithelial cells (IECs) are an important part of the intestinal mucosal barrier (5, 6). Injury to IECs is an important pathological basis for intestinal dysfunction. IECs can produce severe immune stress in a variety of physiological, pathological, dietary, or environmental conditions, and the accumulation of excessive inflammatory cytokines can damage intestinal epithelial cells, resulting in intestinal dysfunction. Therefore, the key to reducing the diarrhea rate and improving the production performance of weaned piglets is to reduce weaning stress and protect the intestinal structure and function of piglets (7). Seeking immunostimulants to promote growth and reduce the intestinal inflammation caused by weaning has become a hot scientific issue.

In recent years, plant extracts as immunomodulators have received worldwide attention due to their nutritional and medicinal potential (8, 9). *Hippophae rhamnoides L*. (sea buckthorn) is a traditional medicinal plant (10). *H.rhamnoides* extracts have shown antioxidant, anti-inflammatory, and anti-viral effects by decreasing cytotoxicity and reactive oxygen species (ROS) generation (11, 12). A recent study showed that *Hippophae rhamnoides* polysaccharide (HRP) protected mice livers from CCl_4_-induced damage through its anti-inflammatory effect and the modulation of the balance between anti-inflammatory cytokines inimmune cells (13). The protective mechanism of chitosan oligosaccharide against LPS-induced inflammatory responses in IPEC-J2 and in mice with DSS dextran sulfate sodium-induced colitis is reported (14). In our previous study, the role of several key regulatory genes and proteins involved in HRP immunoregulation was examined (15, 16). However, the composition and mechanisms of the underlying global regulatory networks at the transcriptome levels are still poorly understood. To confirm the pre-protective effect of HRP on lipopolysaccharide (LPS)-induced intestinal porcine epithelial cells (IPEC-J2) in terms of anti-inflammatory or immunoregulatoryproperties, the pathways enriched by differentially expressed genes (DEGs) should be biologically validated. We employed RNA-sequencing (RNA-seq) to determine the abundance and function of genes (17, 18) and lay the foundation for further study of the anti-inflammatory immune response of HRP in order to provide a theoretical basis and technical support for the treatment and prevention of diseases such as diarrhea caused by intestinal damage in early-weaned piglets.

## Materials and Methods

### Materials

HRP, ≥98% (HPLC) purchased from Nanjing Zelang Biological Technology Co., Ltd., China. HRP was extracted by water decoction and alcohol precipitation as described previously (19, 20). The protein in the filtrate was removed primarily by the Sevage method, HRP was purified on a Sephadex G150 gel (Pharmacia) and the purity was also verified by HPLC. HRP consists of 70% carbohydrate and 14.2% uronic acid. The polysaccharide is composed of mannose, arabinose, glucose, galactose and rhamnose with a ratio of 2.02:1.02:4.24:1:9.22 as the indication of chromatographic analysis of HRP (19).

### Cell Culture and Treatment

The IPEC-J2 cell line source and cells culture method was as described in our previous study (21). HRP was pretreated for 24 h. Subsequently, cells were exposed to LPS for 16 h. The cells were collected, and quickly frozen in liquid nitrogen, and stored at −80°C for future transcriptome analysis. This study included five treatments C represents the control group IPEC-J2 cells without treatment. L represents the treatment group of IPEC-J2 cells induced by 10 μg/mL LPS. H2-L, H4-L, and H6-L represent the pre-treatment of IPEC-J2 cells with 200, 400, and 600 μg/mL HRP, respectively, followed by treatment with 10 μg/mL LPS. The HRP and LPS concentrations used were based on the results of our previous articles (16, 17).

### Total RNA Extraction, cDNA Library Construction, and RNA-seq

According to the manufacturer's instructions, TRIzol reagent was used to extract RNA from the IPEC-J2 cells in different groups. cDNA library construction and RNA-seq were as described by Shao et al. (17).

### Bioinformatics Analysis

High quality clean reads were obtained by removing reads containing adapters, more than 10% unknown nucleotides (N), and low-quality reads containing more than 50% low quality (Q-value ≤ 20) bases. The short reads alignment tool Bowtie2 (22) was used to map reads to the ribosome RNA (rRNA) database. The rRNA mapped reads were removed. The remaining reads were further used in the assembly and analysis of the transcriptome. Gene abundances were quantified by the RSEM software (23). Transcript abundance was normalized to fragments per kilobase of exon model per million mapped reads (FPKM). DEGs between groups were analyzed using the DEGs R package (http://www.rproject.org/). Genes with fold change (FC) >1.2 and a false discovery rate (FDR) <0.02 were considered significant. Significant enrichment of the Kyoto Encyclopedia of Genes and Genomes (KEGG) pathways determined the most important biochemical metabolic pathways and signal transduction pathways that the protein participated in. The KEGG website (http://www.kegg.jp/kegg/) was used to query the immune-related signal pathways involved in significantly differently changed genes, and heat map analysis was performed on the significantly differentially expressed genes involved in immune-related signal pathways. For each treatment group, it is included three replicates to increase reliability of the data.

### Verification of Transcriptome Data Using qRT-PCR

To validate the accuracy of the RNA-seq data, the DEG expressions for the nuclear factor of kappa light polypeptide gene enhancer in B-cells 2 (NFKB2), MAP kinase interacting serine/threonine kinase 2 (MKNK2), mitogen-activated protein kinase kinase 1 (MAP2K1), mitogen-activated protein kinase kinase kinase 8 (MAP3K8), Ras-related protein R-Ras (RRAS), TNF receptor-associated factor 1 (TRAF1), NF-kappa-B inhibitor alpha (NFKBIA), interleukin 8 (IL8), tumor necrosis factor, alpha-induced protein 3 (TNFAIP3), and transforming growth factor beta-1 (TGFB1) were determined by qRT-PCR analyses as described previously (16). We focused on the 600 μg/mL concentration of *H. rhamnoides* polysaccharide to pre-treat the IPEC-J2 cells in the following results (16, 21). The primers for the selected genes are listed in Supplementary Table S1.

### Statistical Analysis

For the qPCR analysis, all data analyses were performed by using SPSS software (SPSS version 20.0, Chicago, IL, USA). Tukey's multiple range test was used to compare the mean values (*P* < 0.05) to indicate significant differences and the results were expressed as mean ± SD.

## Results

### Transcriptome Profiles

The aggregate of 356,912,016 raw reads were accumulated by using the High-throughput RNA sequencing to do the paired-end sequencing of the fifteen constructed libraries. After quality control, assessment of contaminated rRNA and low-quality sequences, 356,207,308 qualified Illumina reads were obtained. Approximately 97.44% of the clean reads were mapped to the reference genome and then used for further gene expression analysis (Table 1). We focused on C, L, and H6-L samples in the following experiment.

**Table 1:** Summary statistics for sequence quality and alignment information of IPEC-J2 cells sample in every group.

**Sample**	**Group**	**Raw reads**	**Clean reads**	**BF_Q30 (%)**	**AF_Q30** ** (%)**	**BF_GC** ** (%)**	**AF_GC** ** (%)**	**Total_Mapped**	**Mapping_** **rate (%)**	**Unique_Mapped**	**Multiple_Mapped**
C-1	C	2,08,95,136	2,08,62,498	93.87	93.99	57.06	57.06	2,03,31,961	97.78	1,97,11,071	6,20,890
C-2		2,10,28,580	2,09,87,072	93.43	93.54	55.14	55.13	2,03,48,751	97.45	1,97,28,826	6,19,925
C-3		2,25,18,532	2,247,2688	93.48	93.61	55.32	55.32	2,17,40,753	97.47	2,10,55,186	6,85,567
L-1	L	2,03,00,292	20262572	93.65	93.79	56.17	56.16	1,95,66,822	97.52	1,88,75,343	6,91,479
L-2		2,37,73,966	2,37,31,954	93.68	93.81	56.09	56.09	2,30,56,064	97.61	2,22,76,195	7,79,869
L-3		2,36,25,640	2,35,82,598	93.51	93.63	55.97	55.96	2,29,31,153	97.66	2,21,51,500	7,79,653
H2-L-1	H2-L	2,11,43,164	2,11,06,066	93.9	94.02	56.49	56.49	2,04,55,400	97.63	1,97,62,846	6,92,554
H2-L-2		2,24,45,906	2,23,96,564	93.19	93.32	56.14	56.14	21629948	97.26	2,09,15,053	7,14,895
H2-L-3		2,46,04,618	2,45,58,736	93.66	93.78	56.04	56.03	2,37,74,066	97.56	2,29,82,487	7,91,579
H4-L-1		2,87,80,540	2,87,16,008	93.13	93.27	56.38	56.38	2,77,37,540	97.33	2,68,17,089	9,20,451
H4-L-2	H4-L	2,45,62,664	2,45,06,630	92.95	93.1	56.62	56.63	2,37,01,504	97.27	2,29,11,180	7,90,324
H4-L-3		2,77,16,820	2,76,60,210	93.49	93.61	55.68	55.68	2,67,26,026	97.32	2,58,29,866	8,96,160
H6-L-1		2,68,24,310	2,67,75,974	93.44	93.56	56.13	56.13	2,58,68,459	97.4	2,50,02,306	8,66,153
H6-L-2	H6-L	2,55,24,164	2,54,70,010	93.45	93.6	56.27	56.27	2,45,00,614	97.06	2,36,43,298	8,57,316
H6-L-3		2,31,67,684	2,31,17,728	93.37	93.5	55.93	55.92	2,23,36,845	97.25	2,15,99,289	7,37,556

*C1–C3 represent the control-group IPEC-J2 cells without treatment, L1-L3 represent the replicates of treatment IPEC-J2 cells induced by LPS with 10 μg/mL, H2-L-1- H2-L-3 represent the replicates of pre-treatment IPEC-J2 cells with 200 μg/mL HRP and followed by co-treatment with 10 μg/mL LPS, H4-L-1- H4-L-3 represent the replicates of pre-treatment IPEC-J2 cells with 400 μg/mL HRP and followed by co-treatment with 10 μg/mL LPS, and H6-L-1- H6-L-3 represent the replicates of pre-treatment IPEC-J2 cells with 600 μg/mL HRP and followed by co-treatment with 10 μg/mL LPS*.

**Figure 1:**
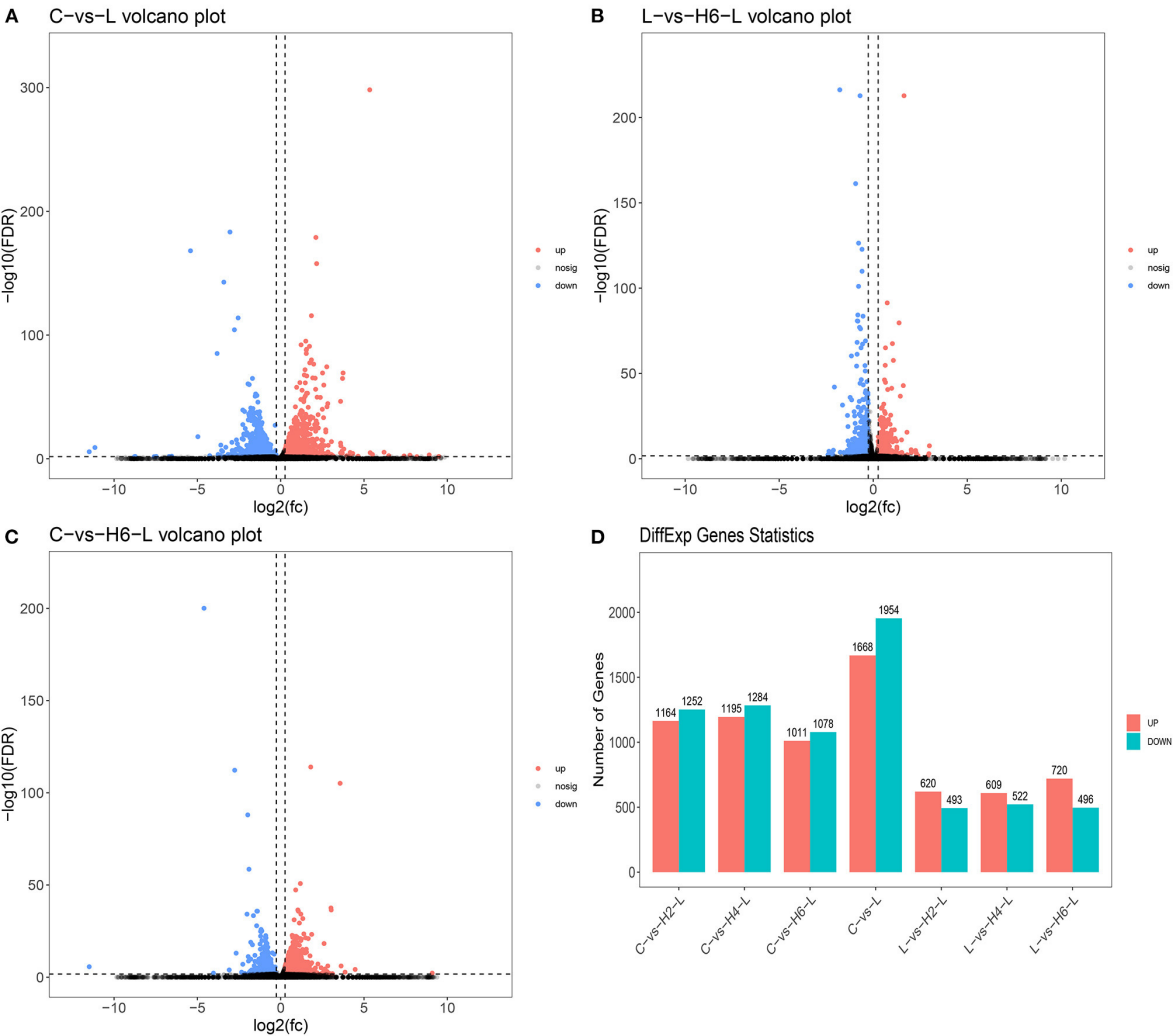
Analysis of the volcano plotof DEGs **(A–C)**. Upregulated genes are shown in red, down-regulated genes are shown in blue, and genes with no significant difference in expression are indicated in black, significance was indicated by *P* < 0.05. DEGs statistics in the comparison group **(D)**.

DEGs were identified using digital gene expression tags. The expression analysis indicated that 3,622, 1,216, and 2,100 DEGs in the IPEC-J2 cells were identified in C vs. L, L vs. H6-L, and C vs. H6-L, respectively (FC >1.2 and FDR <0.02) The most DEGs were identified from C to L (Figures 1A–D and Supplementary Tables S2–S4). These results demonstrated that number of inflammatory cytokines were accentuated after LPS induction. However, number of inflammatory cytokines in HRP pre-treated groups were significantly reduced. The volcano plot shows that HRP plays an important role in the immune regulation of cellular inflammatory damage.

### Functional Enrichment of DEGs

**Figure 2:**
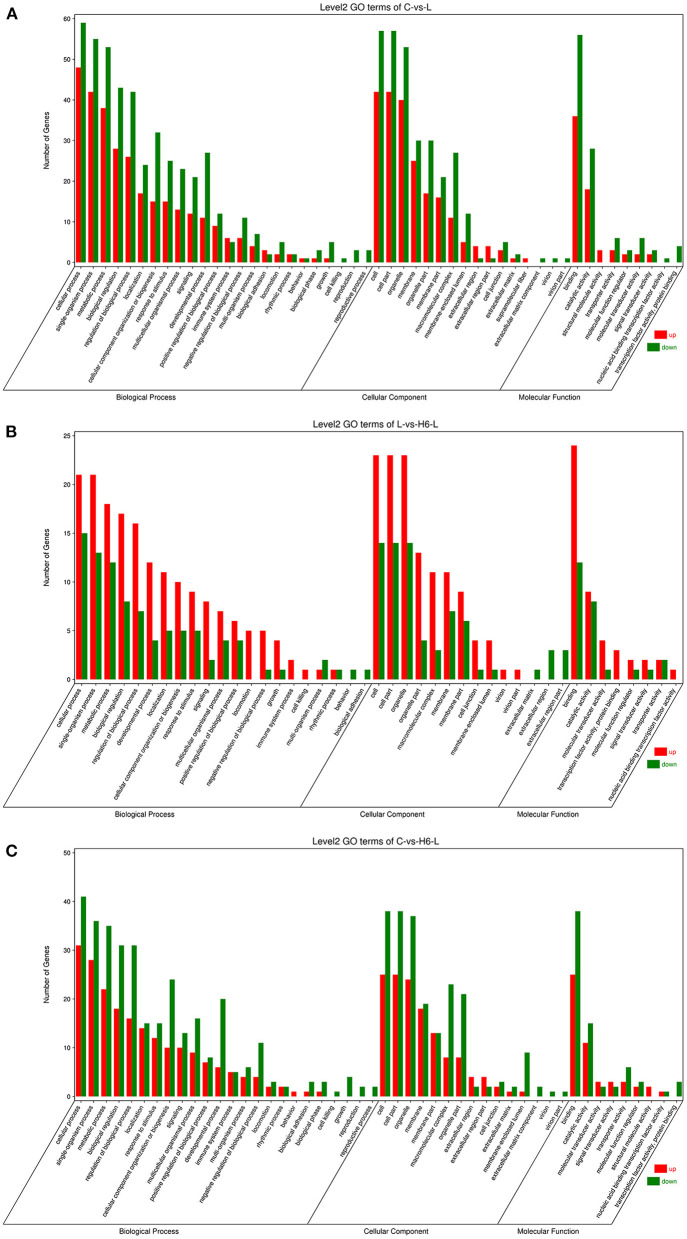
The GO analysis of the DEGs in IPEC-J2 cells. Classification of identified genes based on functional annotations using GO analysis are shown for comparisons between the L treatment and C **(A)**, H6-L treatment and L alone **(B)**, H6-L treatment and C **(C)**.

DEGs were categorized into three Gene Ontology (GO) groups: biological process, cellular component, and molecular function. In the biological process group, many DEGs were categorized as the cellular process, single-organism process, and metabolic process. In the cellular component group, many DEGs were categorized as the cell, cell part and organelle. In the molecular function group, many DEGs were categorized as the binding, catalytic activity, and transporter activity (Figures 2A–C and Supplementary Tables S5–S7).

**Figure 3:**
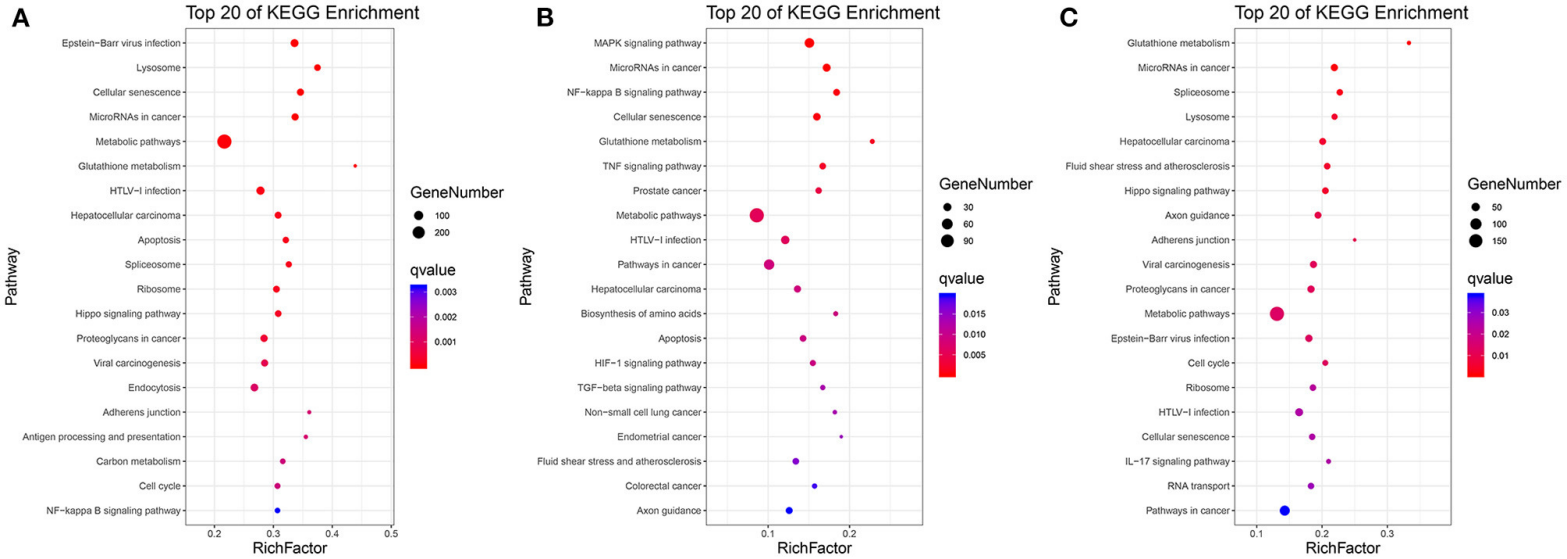
Top 20 pathway enrichment in KEGG pathway analysis. **(A)** C vs. L. **(B)** L vs. H6-L. **(C)** C vs. H6-L. The greater the rich factor, the higher the degree of enrichment. The Q value ranges from 0 to 1 and the closer it is to zero, the more significant.

As shown in Figures 3A–C and Supplementary Tables S8–S10, in C vs. L, 1,442 DEGs were mapped into 322 KEGG pathways. The key pathways were Apoptosis (ko04210) and the NF-kappa B signaling pathway (ko04064). In L vs. H6-L, 531 DEGs were mapped into 307 KEGG pathways. The key pathways were MAPK (ko04010), NF-kappa B (ko04064), TNF (ko04668), Apoptosis (ko04210), HIF-1 (ko04066), and the TGF-beta signaling pathway (ko04350). In C vs. H6-L, 892 DEGs were mapped into 318 KEGG pathways. The key pathway was the IL-17 signaling pathway (ko04657).

### Heat Map of the DEGs Related to Immune Pathways in L vs. H6-L

**Figure 4:**
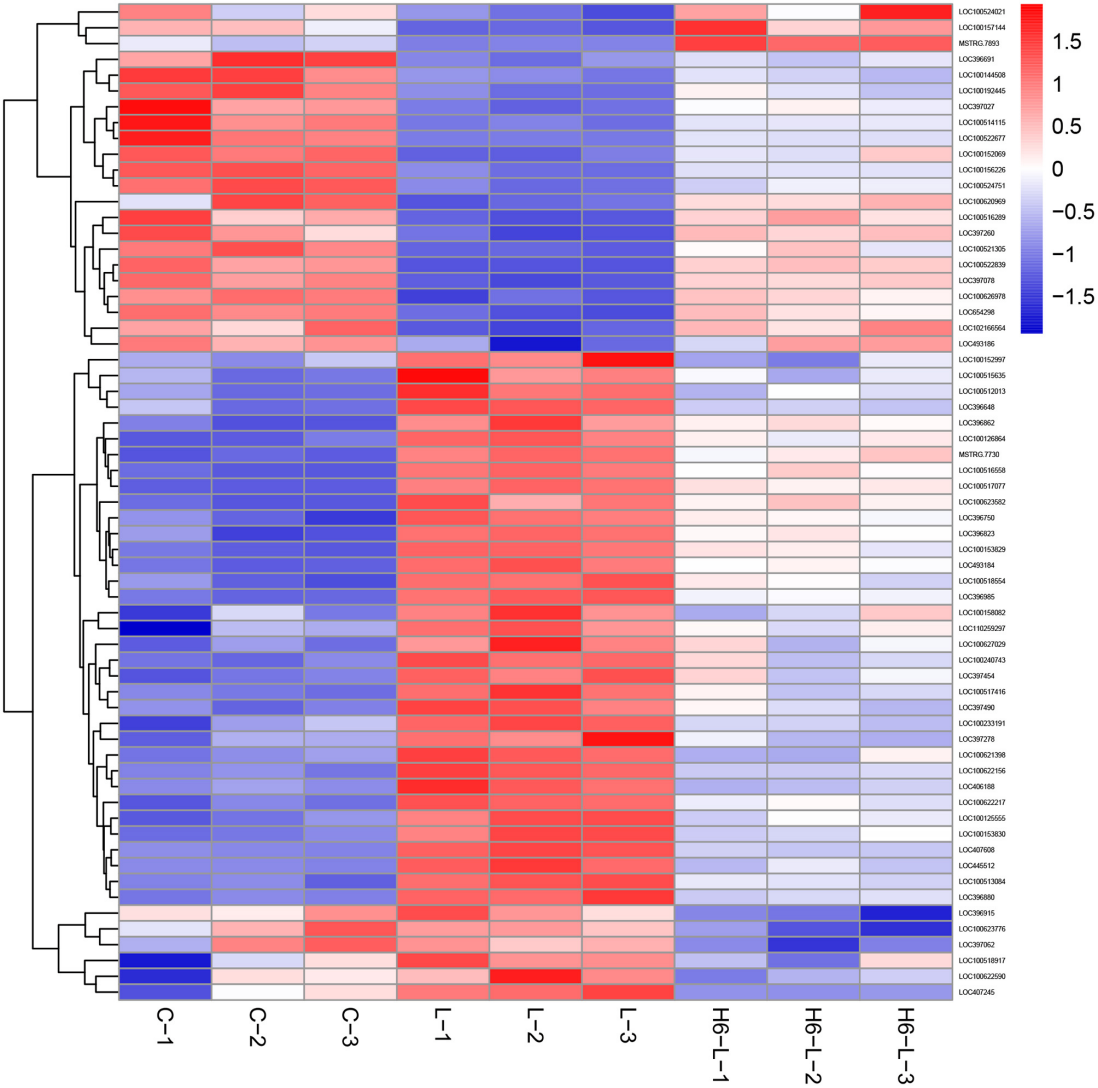
A heat map of the DEGs related to immune pathways in IPEC-J2 cells. The sample gene expression of the C (C1–C3) and L (L1–L3), L (L1–L3) and H6–L (H6-L-1-H6-L-3), C (C1–C3), and H6-L (H6-L-1-H6-L-3) groups is shown using a pseudo color scale, with high levels of expression shown in red and low levels shown in blue.

In Figure 4 and Table 2, the sample gene expression of the C and L, L and H6-L, C and H6-L groups is shown using a pseudo color scale, with high levels of expression shown in red and low levels shown in blue. The results showed that most of the gene expression levels were down-regulated after HRP pre-treatment compared with the LPS group. The Log2-fold change and *p*-value statistics of DEGs related to the main immune pathways in the L-vs.-H6-L group are shown in Table 3. This data indicated that genes were significantly expressed in six immune pathways.

**Table 2:** Significant differentially expressed genes related to the main immune pathways in the L-vs-H6-L comparison group.

**Gene ID**	**Gene** ** name**	**Description**
LOC100153829	NFKB2	Nuclear factor of kappa light polypeptide gene enhancer in B-cells 2
LOC100517077	MKNK2	MAP kinase interacting serine/threonine kinase 2
LOC100233191	MAP2K1	Mitogen-activated protein kinase kinase 1
LOC396648	HSPA1s	Heat shock 70kda protein 1/2/6/8
LOC100158082	PPP3C	Serine/threonine-protein phosphatase 2B catalytic subunit
LOC100152997	GADD45	Growth arrest and dna-damage-inducible protein
LOC100622217	MAP3K8	Mitogen-activated protein kinase kinase kinase 8
LOC100240743	DDIT3	DNA damage-inducible transcript 3
LOC493184	HSPB1	Heat shock protein beta-1
LOC100513084	CSF1	Macrophage colony-stimulating factor 1
LOC100516558	RRAS	Ras-related protein R-Ras
LOC110259297	RRAS2	Ras-related protein R-Ras2
LOC396985	PLAU	urokinase plasminogen activator
LOC100627029	TRAF1	TNF receptor-associated factor 1
LOC406188	NFKBIA	NF-kappa-B inhibitor alpha
LOC100125555	LY96	Lymphocyte antigen 96
LOC100623776	TRIF	Toll-like receptor adapter molecule 1
LOC396750	ICAM1	Intercellular adhesion molecule 1
LOC100153830	MALT1	Mucosa-associated lymphoid tissue lymphoma translocation protein 1
LOC396880	IL8	Interleukin 8
LOC100518917	PARP	Poly
LOC100622156	TNFAIP3	Tumor necrosis factor, alpha-induced protein 3
LOC100622590	BIRC2_3	Baculoviral IAP repeat-containing protein 2/3
LOC100515635	CREB5	Cyclic amp-responsive element-binding protein 5
LOC396915	EDN1	Endothelin-1
LOC397454	ITPR1	Inositol 1,4,5-triphosphate receptor type 1
LOC397278	PMAIP1	Phorbol-12-myristate-13-acetate-induced protein 1
LOC407245	LDH	L-lactate dehydrogenase
LOC396823	GAPDH	Glyceraldehyde 3-phosphate dehydrogenase
LOC445512	HMOX1	Heme oxygenase 1
LOC407608	PGK	Phosphoglycerate kinase
LOC100512013	ALDO	Fructose-bisphosphate aldolase, class I
LOC100126864	IFNGR2	Interferon gamma receptor 2
LOC397062	TFRC	Transferrin receptor
LOC396862	TIMP1	Metallopeptidase inhibitor 1
LOC100623582	NBL1	Neuroblastoma suppressor of tumorigenicity 1
LOC397490	INHBB	Inhibin beta B chain
LOC100518554	INHBE	Inhibin beta e chain
MSTRG.7730	PDGFB	Platelet-derived growth factor subunit b
LOC100517416	DUSP10	Dual specificity phosphatase 10
LOC397260	SMAD2_3	Mothers against decapentaplegic homolog 2/3
LOC100152069	SMAD6	Mothers against decapentaplegic homolog 6
LOC100522839	ID1	DNA-binding protein inhibitor ID1
LOC100522677	E2F4_5	Transcription factor e2f4/5
LOC396691	BMPR1B	Bone morphogenetic protein receptor type-1b
LOC654298	ID2	DNA-binding protein inhibitor ID2
LOC100626978	ID3	DNA-binding protein inhibitor ID3
LOC100144508	ID4	DNA-binding protein inhibitor ID4
LOC100521305	SMAD7	Mothers against decapentaplegic homolog 7
LOC100516289	PFKFB3	6-phosphofructo-2-kinase / fructose-2,6-biphosphatase 3
LOC100156226	EP300	E1A/CREB-binding protein
LOC100192445	PDPK1	3-phosphoinositide dependent protein kinase-1
LOC397027	CAPN1	Calpain-1
LOC493186	SOCS3	Suppressor of cytokine signaling 3
LOC100621398	MRAS	Ras-related protein M-Ras
LOC100620969	EREG	Epiregulin
LOC397078	TGFB1	Transforming growth factor beta-1
LOC100524751	RIPK1	Receptor-interacting serine/threonine-protein kinase 1
LOC100157144	DUSP	Dual specificity MAP kinase phosphatase
MSTRG.7893	NR4A1	Nuclear receptor subfamily 4 group A member 1
LOC100524021	CACNB3	Voltage-dependent calcium channel beta-3
LOC102166564	PDGFRB	Platelet-derived growth factor receptor beta
LOC100514115	FGFR3	Fibroblast growth factor receptor 3
LOC654328	MET	Proto-oncogene tyrosine-protein kinase Met

**Table 3:** Log2-fold change and *p*-value statistics of significant differentially expressed genes related to the main immune pathways in the L-vs-H6-L group.

**Gene ID**	**Gene** ** name**	**Log2 fold change**		***P*** **-value**	
		**C-vs-L**	**L-vs-H6-L**	**C-vs-L**	**L-vs-H6-L**
LOC100153829	NFKB2	1.16	−0.43	9.63E-39	2.65E-18
LOC100517077	MKNK2	1.16	−0.35	5.10E-65	2.88E-39
LOC100233191	MAP2K1	0.61	−0.45	2.23E-12	3.99E-12
LOC396648	HSPA1s	0.82	−0.59	2.61E-19	7.87E-114
LOC100158082	PPP3C	0.48	−0.29	5.85E-07	0.00045
LOC100152997	GADD45	2.28	−2.18	9.91E-06	8.74E-06
LOC100622217	MAP3K8	1.77	−0.76	1.82E-22	5.36E-09
LOC100240743	DDIT3	1.61	−0.76	2.11E-23	8.35E-09
LOC4 93184	HSPB1	1.98	−0.65	4.03E-80	1.82E-68
LOC100513084	CSF1	2.07	−1.01	8.41E-28	1.90E-11
LOC100516558	RRAS	1.40	−0.42	4.76E-52	5.56E-15
LOC110259297	RRAS2	0.61	−0.29	4.70E-12	3.17E-13
LOC396985	PLAU	1.28	−0.57	1.14E-58	1.10E-70
LOC100627029	TRAF1	2.28	−0.88	2.27E-11	0.00050
LOC406188	NFKBIA	0.97	−0.77	4.03E-21	5.22E-21
LOC100125555	LY96	1.79	−0.84	2.07E-16	1.29E-07
LOC100623776	TRIF	0.02	−0.48	0.28430	0.00119
LOC396750	ICAM1	0.82	−0.33	9.75E-18	1.23E-11
LOC100153830	MALT1	1.49	−0.78	1.83E-17	1.33E-08
LOC396880	IL8	1.71	−0.98	4.04E-37	2.07E-18
LOC100518917	PARP	0.40	−0.35	0.00013	0.00022
LOC100622156	TNFAIP3	1.58	−0.99	4.94E-37	4.43E-28
LOC100622590	BIRC2_3	0.50	−0.61	0.00108	1.80E-05
LOC100515635	CREB5	2.53	−1.27	5.95E-17	4.61E-07
LOC396915	EDN1	0.11	−0.68	0.02909	6.20E-11
LOC397454	ITPR1	0.81	−0.39	1.26E-19	2.07E-08
LOC397278	PMAIP1	0.90	−0.69	1.34E-09	1.72E-07
LOC407245	LDH	0.24	−0.32	1.53E-07	3.24E-28
LOC396823	GAPDH	0.71	−0.28	3.78E-19	8.99E-41
LOC445512	HMOX1	2.38	−1.37	6.68E-53	2.62E-28
LOC407608	PGK	0.59	−0.44	4.28E-23	4.92E-58
LOC100512013	ALDO	0.98	−0.60	3.18E-17	2.34E-12
LOC100126864	IFNGR2	1.39	−0.50	9.80E-31	5.24E-10
LOC397062	TFRC	0.03	−0.61	0.07717	8.04E-21
LOC396862	TIMP1	0.81	−0.28	1.02E-16	4.82E-06
LOC100623582	NBL1	1.29	−0.34	1.58E-22	0.00014
LOC397490	INHBB	1.95	−0.99	7.16E-10	6.23E-05
LOC100518554	INHBE	0.80	−0.37	4.79E-15	2.46E-08
MSTRG.7730	PDGFB	1.40	−0.40	1.78E-26	1.14E-05
LOC100517416	DUSP10	2.11	−1.00	5.37E-25	1.01E-10
LOC397260	SMAD2_3	−0.46	0.39	5.08E-06	3.01E-17
LOC100152069	SMAD6	−1.57	0.99	1.65E-13	6.05E-07
LOC100522839	ID1	−1.89	1.64	2.86E-63	3.67E-217
LOC100522677	E2F4_5	−0.90	0.38	7.12E-11	2.86E-06
LOC396691	BMPR1B	−1.08	0.41	2.31E-14	2.66E-05
LOC654298	ID2	−1.34	1.03	3.63E-13	2.60E-12
LOC100626978	ID3	−1.07	0.81	2.45E-19	5.13E-26
LOC100144508	ID4	−1.75	0.66	8.12E-18	0.00041
LOC100521305	SMAD7	−0.97	0.63	7.04E-07	9.90E-05
LOC100516289	PFKFB3	−0.80	0.68	3.84E-06	2.42E-11
LOC100156226	EP300	−1.15	0.52	3.85E-19	3.15E-08
LOC100192445	PDPK1	−0.73	0.31	3.17E-07	0.00189
LOC397027	CAPN1	−0.66	0.36	2.47E-06	1.36E-13
LOC493186	SOCS3	−1.11	0.94	0.00058	1.46E-06
LOC100621398	MRAS	2.52	−1.47	2.76E-06	0.00043
LOC100620969	EREG	−0.76	1.81	2.39E-06	0.00046
LOC397078	TGFB1	−0.56	0.43	2.25E-05	5.57E-13
LOC100524751	RIPK1	−0.81	0.35	1.44E-08	0.00057
LOC100157144	DUSP	−0.26	0.35	0.41187	6.08E-05
MSTRG.7893	NR4A1	−0.29	0.85	0.39207	7.41-17
LOC100524021	CACNB3	−0.27	0.36	0.39343	0.00266
LOC102166564	PDGFRB	−1.20	1.13	0.00016	8.65E-06
LOC100514115	FGFR3	−1.20	0.57	8.41E-21	3.70E-35
LOC654328	MET	−1.04	0.48	5.19E-23	2.23E-17

### Cluster Analysis of DEGs Among the Three HRP Concentrations

**Figure 5:**
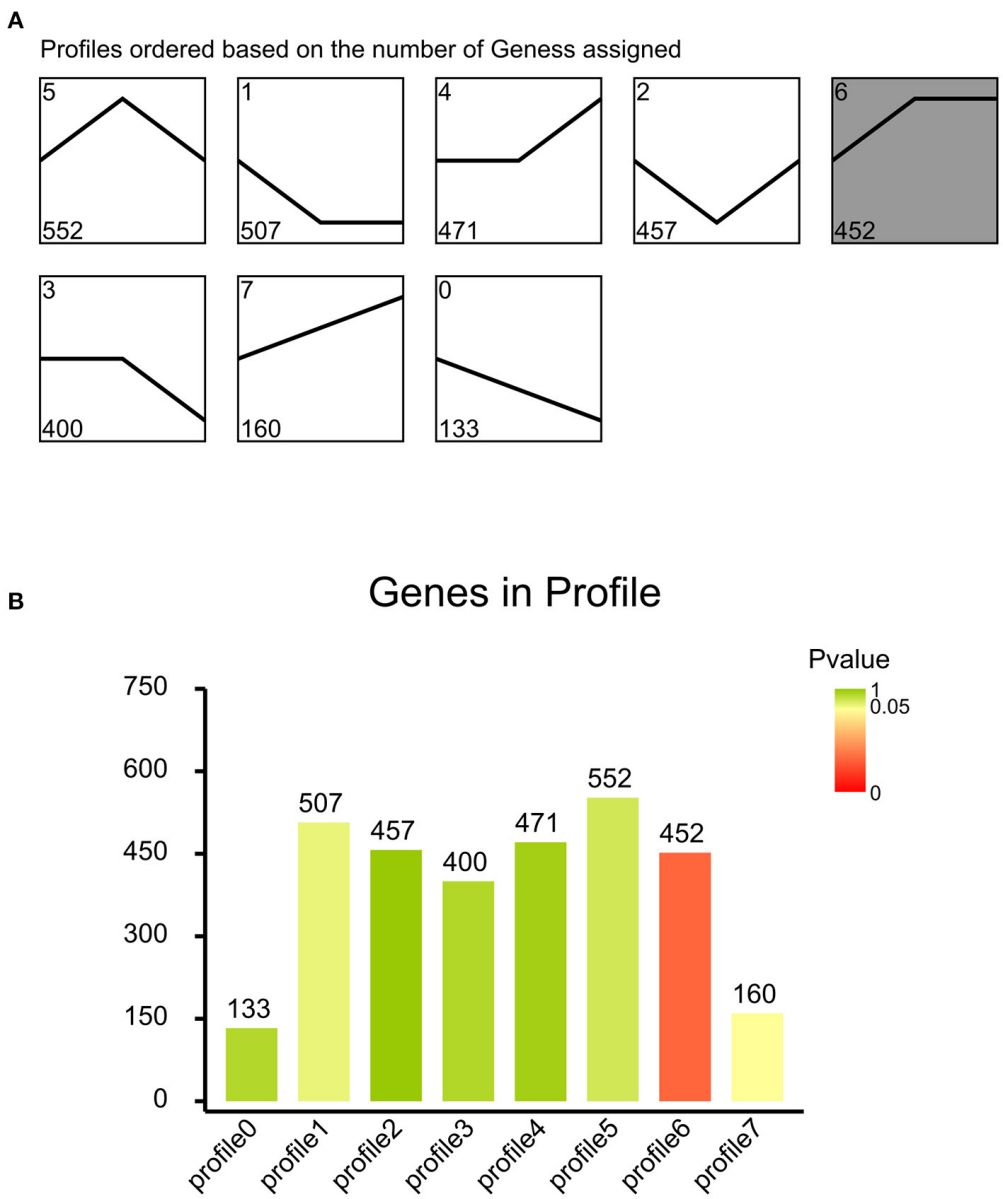
**(A)** Eight profiles of DEGs with unique expression alterations over H2-L, H4-L, and H6-L. The profile number and the number of genes are shown on top of each square. The number of genes assigned is used to order the profiles. The profiles with color (*P* < 0.05): significant enrichment trend. The profiles without color: non-significant enrichment trend. **(B)** Trend DEGs number and *P*-value histogram. X-axis indicates the eight profiles; Y-axis shows DEGs number of every profile. The color of the column represents *P*-value.

**Figure 6:**
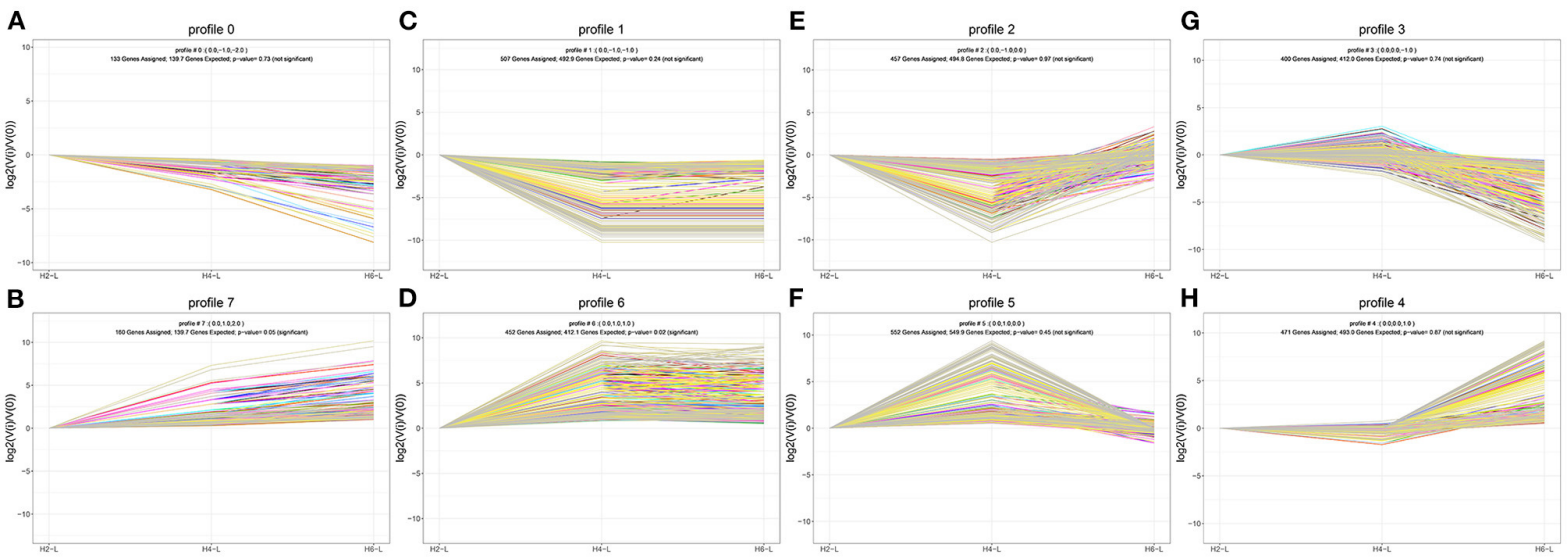
DEGs expression profiles **(A–H)** in the IPEC-J2 cells induced by LPS after HRP pre-treatment. **(A,B)**, **(C,D)**, **(E,F)**, and **(G,H)** are two antagonistic profiles of DEGs curves, respectively. Each X-axis indicates the concentration of HRP pre-treated IPEC-J2 cells (H2-L, H4-L, and H6-L), Y-axis shows expression changes.

To determine the gene expression trajectories, we used the STEM program to categorize the 3,134 DEGs that were differentially expressed in H2-L, H4-L, and H6-L into eight possible expression profiles (*P* < 0.05) (Figures 5A,B, 6A–H, and Supplementary Table S11), in which 612 were clustered into two profiles (*P* ≤ 0.05), including two up-regulated patterns (Profile 6 and Profile 7). Profile 6 and 7 contained 452 and 160 DEGs, respectively. The consistent up-regulation of genes of Profile 6 in H2-L, H4-L, and H6-L indicated that DEGs may contribute to stimulatory functions during the polysaccharide anti-inflammatory function. The up-regulated genes of Profile 7 between only H2-L and H4-L revealed that these DEGs played a key role in the anti-inflammatory process. There was no significant difference in the up-regulated genes between H4-L and H6-L.

### KEGG Pathway Enrichment Analysis of Differentially Expressed Genes Among the Three HRP Concentrations

A total of 11.1% (940/8,499) of the DEGs could be annotated. As shown in Table 4, the metabolic pathways (ko01100), Cytokine-cytokine receptor interaction (ko04060), Neuroactive ligand-receptor interaction (ko04080), Pathways in cancer (ko05200), Olfactory transduction (ko04740), PI3K-Akt signaling pathway (ko04151), Calcium signaling pathway (ko04020), Human papillomavirus infection (ko05165), Jak-STAT signaling pathway (ko04630), and Systemic lupus erythematosus (ko05322) pathways were significantly enriched. The 13 genes among 125 DEGs (22.40%) in Profile 6, and five genes accounting for 11.63% of 43 DEGs in Profile 7 were annotated to the cytokine-cytokine receptor interaction. The three genes among 125 DEGs (2.40%) in Profile 6, and two genes accounting for 4.65% of 43 DEGs in Profile 7 were annotated to the PI3K-Akt signaling pathway. The seven genes among 125 DEGs (5.60%) in Profile 6, and two genes accounting for 4.65% of 43 DEGs in Profile 7 were annotated to the Jak-STAT signaling pathway.

**Table 4:** 10 top KEGG pathways with high representation of the DEGs.

**pathways**	**No. of DEGs with pathway annotation**			**Pathway ID**
	**All profiles** ** (940)**	**Profile** ** 6 (125)**	**Profile** ** 7 (43)**	
Metabolic pathways	162 (17.23%)	28 (22.40%)	5 (11.63%)	ko01100
Cytokine-cytokine receptor interaction	81 (8.62%)	13 (10.40%)	4 (9.30%)	ko04060
Neuroactive ligand-receptor interaction	73 (7.77%)	9 (7.20%)	1 (2.33%)	ko04080
Pathways in cancer	69 (7.34%)	5 (4.00%)	4 (9.30%)	ko05200
Olfactory transduction	67 (7.13%)	8 (6.40%)	3 (6.98%)	ko04740
PI3K-Akt signaling pathway	48 (5.11%)	3 (2.40%)	2 (4.65%)	ko04151
Calcium signaling pathway	42 (4.47%)	5 (4.00%)	1 (2.33%)	ko04020
Human papillomavirus infection	42 (4.47%)	2 (1.60%)	4 (9.30%)	ko05165
Jak-STAT signaling pathway	40 (4.26%)	7 (5.60%)	2 (4.65%)	ko04630
Systemic lupus erythematosus	37 (3.94%)	5 (4.00%)	1 (2.33%)	ko05322

### Validation of RNA-seq Data Using qRT-PCR

**Figure 7:**
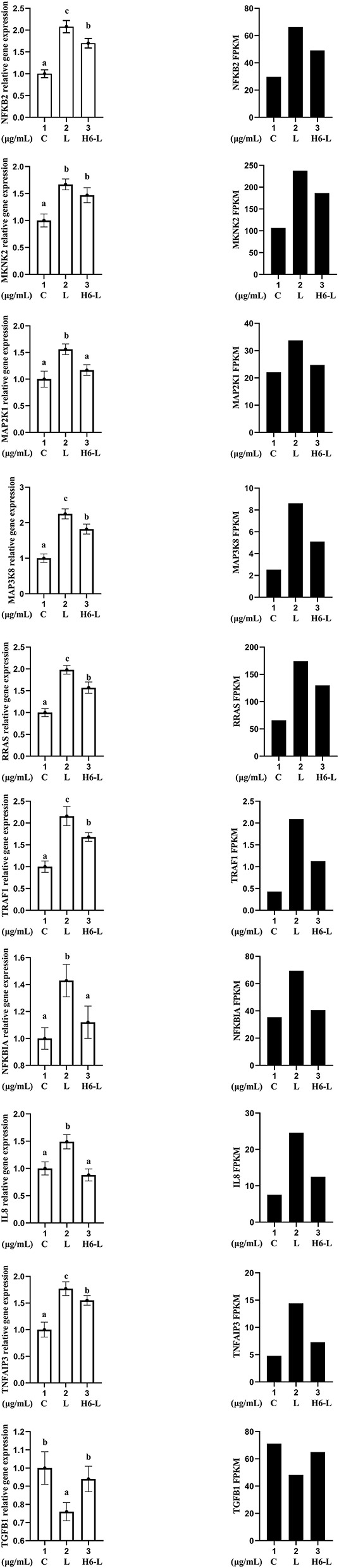
Candidate unigene expression levels revealed by qRT-PCR (left side) and RNA-seq (right side). Data from qRT-PCR are means of five replicates and bars represent SD.

Ten genes (NFKB2, MKNK, MAP2K1, MAP3K8, RRAS, TRAF1, NFKBIA, IL8, TNFAIP3, and TGFB1) were selected for qRT-PCR analysis.The results showed a strong correlation between the RNA sequencing data and the qRT-PCR data (Figure 7). This suggested that the expression results generated by RNA sequencing were reliable.

## Discussion

In the current piglet production system, early weaning is an important means to improve the efficiency of pig production (24, 25). However, early weaning is very stressful to piglets, and can easily cause piglet immune stress and affect the healthy growth of piglets. How to alleviate the immune stress of piglets has become an area of concerning the piglet industry (26, 27). *Hippophae rhamnoides* extracts are widely used to enhancing immunity in both healthy and diseased animals (28). Polysaccharide is the main active ingredient of *H.rhamnoides*. HRP has been shown to have immunomodulatory effects (10). No research has been conducted on the molecular mechanism of HRP in piglets by transcriptome sequencing. Variation in gene expression may provide a key to uncovering the mechanisms of diseases. Transcriptome sequencing, also called RNA-seq, provides a new technique to quantify whole-genome expression profiling in any organism. It promises digital transcriptome profiling with high resolution and is rapidly replacing microarray technology (29, 30). Studies have used RNA-seq technology to explore the protective mechanism of IPEC-J2 cells stimulated by LPS after astragalus polysaccharide (APS) pretreatment. APS relieves cell damage by inhibiting the activation of the MAPK and NF-κB inflammatory pathways, thereby reducing intestinal inflammation (31). Cluster analysis revealed 3134 DEGs that were differentially expressed in H2-L, H4-L, and H6-L into eight possible expression profiles, in which 168 were clustered into two profiles. The up-regulated genes of Profile 6, only between H2-L and H4-L, revealed that these DEGs played a key role in the anti-inflammatory process. The consistent up-regulation of genes of the Profile 7 in H2-L, H4-L, and H6-L indicated that DEGs may contribute to stimulatory functions during the polysaccharide anti-inflammatory process. KEGG enrichment analysis found that the six identified pathways were related to the immune system. Among the six identified pathways related to the immune system, the MAPK signaling pathway and NF-κB signaling pathway play important immunomodulatory roles in our study. Finally, we selected 10 DEGs (NFKB2, MKNK2, MAP2K1 (MEK1), MAP3K8, RRAS, TRAF1, NFKBIA, IL8, TNFAIP3, and TGFB1) related to the main immune pathways to validate the RNA-Seq data using qRT-PCR.

The downstream signal transduction pathways mediated by LPS mainly include the NF-κB signal transduction pathway and the MAPK signal transduction pathway (32). Extracellular regulated protein kinases (ERK), c-Jun N-terminal kinase (JNK), and p38 mitogen-activated protein kinase (p38 MAPK) belong to the three subtypes of the MAPK signaling pathway (33). The Ras/Mitogen-activated protein kinase kinase (MEK)/ERK pathway is one of the most important signal transduction pathways among the MAPK pathways. The Ras/MEK/ERK pathway involves the regulation of a variety of physiological functions of cells and plays a key role in the pathogenesis and pathophysiology of various diseases (34). The activation of ERK is a key step in transferring signals from surface receptors to the nucleus. The activation of ERK induced by LPS leads to the secretion of large amounts of tumor necrosis factor-alpha (TNF-α), as well as interleukin-6 (IL-6) and IL-8, and increases the expression of inducible nitric oxide synthase and nitric oxide. Son of Sevenless (SOS) binds to RRAS-Guanosine diphosphate (GDP), prompting guanosine triphosphate (GTP) to replace GDP on RRAS and activate RRAS protein, then activate MEK and ERK sequentially. In recent years, some initial reports on targeting SOS to inhibit the activation of RRAS, thereby inhibiting the activation of the MAPK signaling pathway, have also achieved satisfactory results (35). The activation of ERK promotes the secretion of MKNK. MKNK is an important downstream protein kinase of ERK that has an important immunomodulatory function. MKNK dysfunction can inhibit the inflammatory signal of upstream ERK and affect downstream eIf4E and CREB and other effector proteins, thereby preventing cell inflammation. MAP3K8 is essential for the activation of the intracellular MAPK/ERK pathway induced by LPS in cells (36). Therefore, MAP3K8 is a critical factor for the production of pro-inflammatory cytokines during immune responses (37). Therefore, in our study, the results showed that MKNK2, MAP2K1, MAP3K8, and RRAS gene expression levels were down-regulated after HRP pre-treatment compared with the LPS group. The reduction of MKNK2, MAP2K1, MAP3K8, and RRAS gene levels plays an important immuno-regulatory role in HRP alleviating LPS-induced cell damage.

LPS, a trigger of inflammation, can activate the NF-κB signaling pathway (38). NFKBIA is a specific inhibitor of NF-κB that binds to NF-κB at a resting state to cause NF-κB to enter an inactive state. Phosphorylated NFKBIA is separated from NF-κB, and NF-κB is activated. Activated NF-κB migrates to the nucleus, where NF-κB nuclear transcription factor can up-regulate the levels of inflammation-related genes TNF, IL-6, and IL-8 and down-regulate the level of TGFB1 (39). Studies have proven that LPS promotes the degradation of NFKBIA, activates the DNA binding ability of NF-κB, and regulates the gene expression level of cytokines (40). Moreover, TRAFs are key regulatory proteins in NF-κB signaling pathways. TRAF1 enhances the activation of TNF-R2 induced by NF-κB (41), therefore promoting the release of a large number of inflammatory cytokines. A previous study showed that TRAF1 is over-expressed in a variety of lymphoma and leukemia cell lines and is a crucial mediator of diverse oncogenic signaling in the development of lymphoid malignancies. TNFAIP3 is a cytokine-induced protein that inhibits apoptosis and activates NF-κB (42). The main function of TNFAIP3 is to inhibit the activity of NF-κB and inhibit TNF-mediated apoptosis, thereby having an important impact on immune regulation and inflammatory processes (43, 44). The release of NFKB2, TRAF1, NFKBIA, IL8, and TNFAIP3, as important regulatory genes of NF-κB, can activate the upstream pathway NF-κB. Jayashankar et al. found that the intervention of supercritical carbon dioxide extract from seabuckthorn leaves can inhibit the expression levels of TNF-α and IL-6 after LPS-induced inflammatory damage, inhibit the activation of the MAPK/NF-κB signaling pathway, reduce inflammation, and play a immunomodulatory role (45). Our study indicated that NFKB2, TRAF1, NFKBIA, IL8, and TNFAIP3were increased and TGFB1 was reduced after LPS induction. However, after HRP pre-treatment, the gene expression level showed the opposite trend and they played an important immune-regulatory role in HRP alleviating LPS-induced cell damage, which provided more targets and prevention directions for theoretical and basic research on intestinal health. Studies have shown that APS may block radiation-induced bystander effects (RIBE) in bone mesenchymal stem cells (BMSCs) induced by the irradiated A549 through regulating the MAPK/NF-κB pathway (46).

## Conclusions

This study was the first using a RNA-Seq technique to establish a dynamic transcriptomic profile of three stages (C, L, and H6-L) related to pre-treatment with HRP followed by challenge with LPS in IPEC-J2 cells. Subsequently, bioinformatics analysis (GO, KEGG, and series cluster) helped us to identify key regulatory genes (IL8 and NFKB2, among others.) related IPEC-J2 cellular immune regulation. Transcriptome analysis also showed that HRP protected IPEC-J2 cells from LPS-induced inflammation and decreased the expression of inflammatory cytokines by mainly inhibiting the MAPK/NF-κB signaling pathway. This study does not only provide the useful transcriptomic reference for HRP to effectively protect LPS-induced inflammatory damage in IPEC-J2 cells, but also provides a benchmark for the discovery of biomarkers related to HRP immune regulation.

## Data Availability Statement

The data presented in the study are deposited in the following repository: https://www.ncbi.nlm.nih.gov/Traces/study/, accession number PRJNA854604.

## Author Contributions

ML, HS, and LZ contributed to conception, design of study, drafting the manuscript, and critical revision. YZ conducted acquisition of data. LC conducted analysis of data. All authors read and approved the final manuscript.

## Funding

This project was supported by grant from the National Key Research and Development Program of China (2017YFD0500506) and the Personnel Foundation of Heilongjiang Bayi Agricultural University (NO. XYB202015).

## Conflict of Interest

The authors declare that the research was conducted in the absence of any commercial or financial relationships that could be construed as a potential conflict of interest.

## Publisher's Note

All claims expressed in this article are solely those of the authors and do not necessarily represent those of their affiliated organizations, or those of the publisher, the editors and the reviewers. Any product that may be evaluated in this article, or claim that may be made by its manufacturer, is not guaranteed or endorsed by the publisher.
